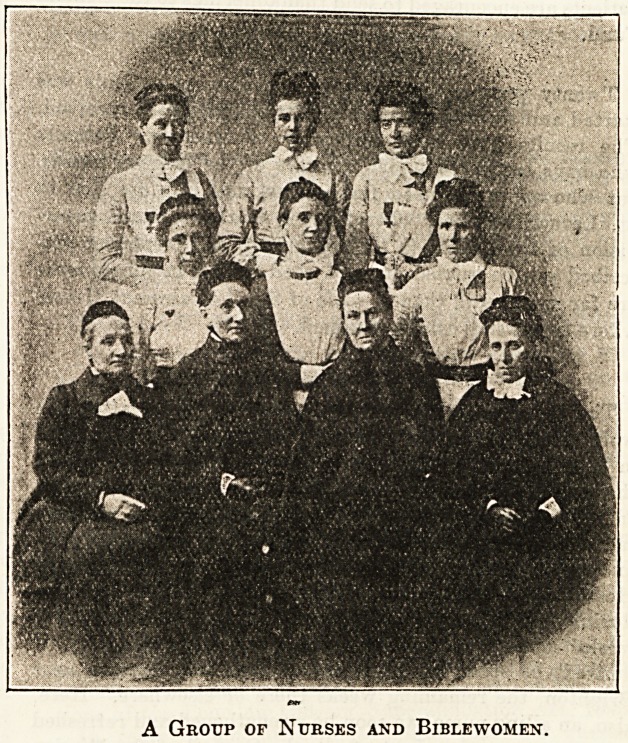# "The Hospital" Nursing Section

**Published:** 1906-06-02

**Authors:** 


The Hospital.

Nursing Section.

Contributions for "THE HOSPITAL," should be addressed to the EDITOR, "THE HOSPITAL" NURSING SECTION, 28 & 29 Southampton Street, Strand, London, W.C.

No. 1,027.—VOL. XL. SATURDAY, JUNE 2, 1906.

### Notes on News from the Nursing World.

### MEETING AT THE COLONIAL OFFICE.

THERE are two special features of interest in connection with the annual meeting of the Colonial Nursing Association, which will be held on the afternoon of Wednesday, June 13. One is that it will take place at the Colonial Office; and the other that the speakers will include the Colonial Secretary, Lord Elgin, by whose permission, of course, the supporters of the organisation will have the privilege of assembling in the Library of the Colonial Office, which is approached by the entrance in Downing Street, has thus shown his keen desire to promote a movement largely affecting his own department. His speech, and that of the Chairman, Lord Ampthill, whose name is associated with a useful nursing institute in India, will be sure to command attention.

### ASYLUM WORKERS AND PENSIONS.

THE annual report of the Asylum Workers' Association, to be presented at the meeting on Friday afternoon, Sir John Batty Tuke, M.P., M.D., in the chair, shows that the finances continue in a sound condition, though the number of members is slightly less, being 3,227, as against 3,235 at the end of 1904. The credit balance was, however, £93 17s. 7d., as against £81 17s. Mention is made of the fact that last year, for the first time since the institution of the medals, there was no female applicant for the gold medal, and consequently a second gold medal was awarded to a male candidate. There is a reference of some length to the claims of asylum workers for assured and adequate pensions, and it is stated that 64 members of the new Parliament, including two Cabinet Ministers, have pledged themselves to support any proposal dealing satisfactorily with asylum superannuation. In this, as in many other matters, we agree that the influence of an association representing the rank and file of asylum workers cannot fail to be of weight with the authorities.

### NIGHT-NURSING IN POOR-LAW INFIRMARIES.

AT the ceremony attending the opening of the new wing of Keynsham Poor-law Infirmary, Mr. E. H. Wethered, Local Government Board inspector, was one of the speakers. In the course of his remarks he dwelt on the improvements which have taken place in the conditions of nursing in poor-law institutions during the last ten years. He went on to emphasise the importance of adequate night nursing. Until lately it had been very largely done by paupers, and so recently as January 1, 1899, there were only 43 night nurses throughout the whole of his large district. At the present time there are 82. He was satisfied not only that these night nurses must be a very great comfort to the sick, but that the ratepayers do not grudge the expense incurred in that way. We do not think they do. But in any case it is essential that there should be night nurses, instead of pauper attendants, in charge of the sick, and we regret that the Nursing Order of 1897, which prohibits pauper nursing, is not always enforced as it ought to be.

### ARMY NURSING IN AMERICA.

AT the end of three years the names of 100 nurses have been enrolled on the volunteer list of the Army Reserve Corps in America. Of this number it appears that 36 have given previous service, and that the remainder are post-graduates in different parts of the United States. The situation, considering the length of time which has elapsed since applications were invited, does not suggest enthusiasm, but it is only fair to note that serious causes for this apparent lack of patriotism have been alleged, and that these allegations have not been challenged. A nurse whose name is one of the hundred enrolled on the list, and who therefore cannot herself be accused of want of patriotism, affirms that the disposition of women to respond to the call to join the Army Reserve Corps may be due, in part, to the unsatisfactory manner in which members of the Army service have been treated in the past. Reciting her own experience, she mentions that when she was chief nurse of a small military hospital in Northern Luzon, the commissary officer complained that in respect to ration it was hard to class the nurses properly, and proceeded to rank them with mule-drivers! This sentiment, she says, seems to be that of the army as a whole, although many are too polite to express it. But in spite of her conclusion, and notwithstanding her dissatisfaction with the position assigned to the army nurses in the United States, she recently begged them not to allow any sense of injustice to affect their patriotism. Probably her creditable appeal has had something to do with the increase of recruits from 50 to twice that number in a few months.

### NURSING EUROPEANS IN INDIA.

IT cannot be said that the Up-country Nursing Association for Europeans in India makes rapid progress. The strength of the nursing staff in December 1905 was the same as at the end of 1904, twelve nurses being on duty at the end of each year. This is but a small number bearing in mind the fact that the organisation has been established for twelve years, and in the annual report just issued, it is stated that a larger income is wanted to enable it to maintain an adequate staff of nurses in Northern India and to extend the work to new centres if the opportunity occurs. The average annual income from subscriptions and donations for the past five years has only amounted to £146, which is barely sufficient to meet the expenses of sending out two nurses a year. The Committee believe that work could be found in India for five, which would mean increasing the staff to fifteen.

### ANSWERING QUESTIONS IN WRITING.

The report of the examination of probationers of Portsmouth Poor-law Infirmary, by Dr. John Fawcett, of Guy's Hospital, is satisfactory, except on two points. The examiner states that the answers were not so detailed as they might have been, and that the replies to two simple practical questions which he set at the end of the first year paper were disappointing. He adds, however, that the *viva voce* examination showed that the nurses actually possessed a greater knowledge of the questions than they had shown in their papers. This we can quite believe. Some very capable nurses fail to express themselves clearly in writing, just as others who are not so capable write with ease. The importance of being able to answer questions in writing must not, however, be under-estimated; and such defects as the examiner points out in this instance can be remedied by application and perseverance. One of the objects of our examination questions is to encourage nurses to practise writing, and the moral of the Portsmouth report is that they need to do so.

### DISTRICT NURSING FEES.

A point, on which we have often commented, was brought out at the annual meeting of the Norwich District Nurses' Association, which has lately been affiliated to Queen Victoria's Jubilee Institute. One of the medical men in his speech mentioned that 68 of his patients had been attended by the nurses during the past year, and in every case the nursing had been satisfactorily carried out. At the same time he entered a protest against the poor patients being asked for the fee of a shilling. This plan, he believed, was disliked alike by medical men, nurses, and patients; and it was when they were in health, not in sickness, that the working classes should be asked to contribute to the Association. Miss Amy Hughes, in her address, endorsed this opinion, and argued that the poor might with advantage be encouraged, on the lines of a benefit club, to contribute a minimum of 2s. a year to the funds, but that the Jubilee Institute deprecated payment of the nurse by the visit or at the time of illness when the patients wanted all their money for the doctor's fees and for extra nourishment.

### THE LATE MISS ALICE PHILLIMORE.

In his address to a large congregation of nurses at St. Paul's Cathedral on Tuesday, Canon Scott Holland referred in appropriate terms to the loss sustained by the nursing world in consequence of the death of Miss Alice Grenville Phillimore, who, he said, had been largely instrumental in making the gathering at St. Paul's a possibility and a success. It is not only at the committee meetings of the East London Nursing Society, which she regularly attended, that her presence will be missed. For many years Miss Phillimore took an active personal interest in nursing matters. In 1896, when Gloucester was devastated by small-pox, she was a volunteer helper, and, attached to the band of clever sisters, did magnificent work in the city, often assisting with her own hands to carry the dead out of the hospital and to lay them in their coffins. She rendered valuable help to the East End Mothers' Home, and was joint administrator with her cousin, Miss Denison, of the Ossington Nursing Trust, a fund established by her aunt, the late Viscountess Ossington, for the nursing of the sick and poor in Great Britain. Miss Phillimore initiated at the Royal Berkshire hospital, at that time in urgent need of funds, a Ladies' Linen League on the lines of one previously formed at the Westminster Hospital; she provided a district nurse for the parish of Shiplake and also for Dunsden, among her own people, and herself supervised the work; and during the South African war she constantly took part in the distribution of the fund for the wives of the soldiers and sailors. Her activities were boundless, and her large-heartedness, no less than her generosity and common sense, endeared her to all who came in contact with her.

### KINGS' COLLEGE HOSPITAL FETE.

In addition to our report of the opening of King's College Hospital Fête, it may be mentioned that after the first day the stall in charge of the nursing staff of the hospital expanded greatly. One bunch of yellow malmaison carnations deserved the attention they received, for they were specially forced for the occasion by a friend of Mrs. Headlam. As the verdict of the Covent Garden market people was that they could not produce such flowers themselves anywhere, the price of 2s. each was not exorbitant. A pretty little group near the vegetable stall, which attracted considerable attention, two babies from the hospital in charge of a nurse, did their best to help in the good cause. A small boy sold button-holes, and a tiny mite hugged a grey kitten with so much affection that it would have seemed hard to deprive her of it. A good report was received from the Army nurses' stall. At Lady Methuen's stall a very handsomely carved chest, the work of soldiers at Shorncliffe, presented by Lord Methuen, was a feature.

### MR. PAWLING'S HOME.

Those of our readers who live in the North of London and have time to spare might like to associate themselves with Mr. Sydney Pawling in establishing a new hospital for children at Hadleigh Green. Mr. Pawling has the support of several hospital surgeons, including Mr. H. H. Clutton, of St. Thomas's; Dr. Warrington Haward, of St. George's; Dr. Eric Pritchard, and the committees of the Great Ormond Street Children's Hospital, the Evelina Hospital, and the Invalid Children's

Aid Association. All of them testify to the existence of an urgent demand for the treatment of surgical cases in a home hospital, and we hope that little difficulty will be experienced in raising the necessary funds to establish the enterprise upon a sound financial basis.

### SCHOOL NURSES.

Speaking last week at a meeting of the "Hampstead Health Society," Mr. Newsholme, medical officer of health for Brighton, alluded to the great benefit, which is already making itself felt, of the medical inspection of schools, and warmly eulogised the work of the nurses in the primary schools where they have been appointed. Their influence on the general hygiene and personal cleanliness of the children—an influence which must inevitably extend to their homes—could not, he said, be too heartily appreciated, and he hoped that the day was not far distant when there would be no County Council school without its nurse.

### A MEMORIAL TO A PIONEER.

It is none too often that the work of pioneers is recognised, and we are glad to learn that warm support is being afforded to a movement for a memorial to the late Mrs. Cash, of Hampstead, who died a short time ago. Mrs. Cash resided for a quarter of a century in Hampstead, and as far back as 1883 provided a trained nurse for the sick poor in their own homes free of cost. A year later, as the direct result, the Hampstead Nursing Association was formed, and the development of the organisation has been largely due to the generous assistance of Mrs. Cash, who annually contributed a larger sum than the salary of a single nurse. The committee which has been formed to promote the memorial, with Sir George Barham as Chairman, propose to raise a fund which, it is hoped, will be sufficient to invest and form a nucleus of income to be devoted to the object in which Mrs. Cash was so keenly interested. For this proposal a considerable sum is needed, but there are no doubt among the numerous friends and admirers of the deceased lady many who will rejoice to have the opportunity of doing honour to her in the way she would have most appreciated.

### PROPOSED SURGICAL HOSTEL FOR GENTLEWOMEN.

A meeting was held on Tuesday at Lauriston House, Wimbledon Common, to discuss the projected establishment of a new surgical hostel for gentlewomen. The chair was taken by Sir Wroth Lethbridge, and the speakers included Dr. Norris Wolfenden and Miss Wortabet, who is well known in the nursing world. Although the scheme has only assumed definite proportions during the last three or four months, it has already many influential supporters, and as soon as sufficient funds are forthcoming it is hoped that a house overlooking Regent's Park, which has been inspected and found suitable, may be secured. The hostel is to accommodate 100 patients; and is intended for the wealthy, for people with a limited income, to whom the fees, inclusive of doctors and nursing, will not exceed £3 3s.; and for a few deserving gentlewomen unable to pay any charges at all, who will be treated free. It is confidently asserted by the promoters that after the initial £20,000 has been raised there will be no need of any further appeal to the public, as in their opinion the institution will become self-supporting.

### DEVONSHIRE IMITATING PLAISTOW.

An influential Committee is engaged in perfecting a scheme initiated by Countess Fortescue and the Earl of Mount Edgcumbe to provide nurses for the poor in Plymouth and Devonport, and to establish a training home of a character similar to that at Plaistow. At a meeting of the Provisional Committee donations amounting to £245 were reported, including two anonymous gifts of £100 each, and £25 from the Earl of Mount Edgcumbe. The association will be affiliated to the County Association and Queen Victoria's Jubilee Institute. One nurse is already at work.

### SPLENDID BEQUEST TO A NURSING INSTITUTE.

By the will of the late Mr. W. Rae, of Northampton, the Queen Victoria Jubilee Nursing Institute in that town benefits to the extent of no less than £5,000. During his life Dr. Rae took the warmest interest in the Institute, and he has enriched it at his death with a splendid bequest which will enable the managers to extend the sphere of its operations, and give occasion to many poor people in Northampton to feel grateful to him.

### BRENTFORD UNION INFIRMARY.

The final examination for nurse probationers in their third year of training was held at the Brentford Union Infirmary, Isleworth, last month. Nine nurses were successful in passing and obtaining a high percentage of marks. Mr. Stephen Paget in his report expressed the "pleasure it was to him to examine the nurses, and to observe how keen and careful and accurate they were in their work. He thought that the whole system of the nursing was good, and that the probationers were thoroughly trained and taught." The nurses, who have passed their final examination in medical and surgical work, can now elect to be more fully instructed in massage and be prepared for the examination of the Incorporated Society of Trained Masseuses. Candidates from amongst them will be selected to fill vacancies as pupil midwives in the lying-in wards and be prepared for the examination held by the Central Midwives Board. They will also be given some experience in a ward sister's duties.

### SHORT ITEMS.

The foundation-stone of the University College Hospital School of Advanced Medical Studies, Nurses' Home and Maternity Students' Home, will be laid by Sir Donald Currie on Monday, June 11, at 4 p.m.—For the post of matron of Salop Infirmary, Shrewsbury, to which Miss Appleyard has just been appointed, there were 93 applications.—A Queen's nurse has been engaged for the parish of Holy Trinity, Upper Tooting, under the auspices of the new vicar, Dr. Lindesay.

### The Nursing Outlook.

"From magnanimity, all fears above;
From nobler recompense, above applause,
Which owes to man's short outlook all its charm."

### A FEMALE QUACKSALVER, "LATE OF ST. BARTHOLOMEW'S HOSPITAL."

THE quacksalver is a familiar figure in human history, and amidst the various impostors who have preyed on the folly and credulity of mankind occupies a position of notorious and nauseous prominence. No age or race has been exempt from his lying tongue and brazen face, and none has failed to contribute to the tale of his victims. The twentieth century finds him still with us, and still, as of yore, he numbers his crowds of followers and admirers. Nor have his methods undergone much change. Shameless imposture, a wholesale disregard of the truth, impudent claims of miraculous or semi-miraculous power, combined with a keen eye for certain human frailties, form, as ever, his stock in trade, and with the aid of these he succeeds, only too often, in filling his pockets at the expense of the particular community which happens to support his parasitic existence. In his most contemptible form his selfish aims and unscrupulous methods are concealed beneath the cloak of philanthropy and religion, and here there results that peculiarly vile creature in whom the quack and the hypocrite are successfully fused.

No past disasters or individual exposures will protect fools against a love of the suave voices of knaves and impostors, and had we nothing to our hand other than general truths we should have utilised our space for some more hopeful crusade. But where "wise saws" fail, success will sometimes adhere to "modern instances." One of these—and one, moreover, of a peculiarly revolting type—has recently been brought to our attention, and as it closely concerns the constituency to which we speak in these columns, there is a reasonable claim that it should here be dealt with as a matter related to the honour and good name of the nursing profession. Further, as will be seen later, the case involves the responsibility of a great hospital and nursing school, not to speak of the protection of the public and of those engaged in an honourable calling.

The particular and individual quack whom we have now to introduce to our readers is a person who proclaims herself as "Nurse Argenti." From a printed circular at present lying before us, and which is, we understand, used for house-to-house distribution, we learn that this person is "a specialist in all complaints peculiar to her sex," that "she is anxious for cases that have been given up as incurable," and that she "is endowed with a wonderful power, and all who come under her treatment seem to get well, as if by magic." These are, of course, familiar protests, but their special significance to us is provided by the fact that they are offered by a person who claims to have had 20 years' experience as a hospital nurse, and announces herself as "late of St. Bartholomew's Hospital, London." Whether this latter statement is accurate or inaccurate is not at this moment a matter of prime concern. The essential question is whether or not the hospital affected is able to put a stop to the association of its name with such impudent and audacious pretensions. The statements above quoted ought to be sufficient in themselves to rouse any self-respecting institution into action; but worse remains to be told. The usual claims of the quacksalver are here reinforced by what is nothing less than gross blasphemy. This "Nurse Argenti," announced as "late" of the greatest of our metropolitan medical schools, has the courage to tell the public that "when I left the hospital I received a divine inspiration" and that "in every case I undertake I have God's guidance." Following immediately on these impious statements she calls attention to the "Malthusian question," and offers the use of certain secret remedies by which a "youthful appearance" may be maintained. Still more, she describes herself as a "specialist in all cases of irregularities, stoppages, etc.," and invites the public to "call and see samples" of the "latest improved specialities in india-rubber." In short, the woman, on her own showing, is cultivating a class of business which cannot even be defined in public print, and which is a notorious outrage on common decency and on social and individual well-being; and with all this she advances a pretence of Divine guidance and inspiration!

Some care and respect for public morality might well invoke an attempt to put a stop to the whole of these shameless and disgusting proceedings. It is, however, possible that they escape a technical breach of the law of the land. But surely it cannot be possible that the authorities of St. Bartholomew's Hospital are helpless to prevent the prostitution of the good name of the great and noble institution over which they preside. At present this is being used to lend a colour of professional efficiency and personal honour to the practice of indecent and outrageous quackery, and it lies with those whose duty it is to guard it from stain to take prompt and decisive action. This is urgent also on public grounds. Some of the most ignorant and most helpless members of the community are being made the dupes of a quack who masquerades under the repute of this great hospital. And every hospital nurse suffers prejudice from the same performance. The demand for action is imperative. If the existing law affords no help, then resort must be had to the law-makers.

### The Care and Nursing of the Insane.

By Percy J. Baily, M.B., C.M.Edin., Medical Superintendent of Hanwell Asylum.

### I.—ANATOMY AND PHYSIOLOGY.

(*Continued from page* 105.)

*The Throat or Pharynx* is that part of the alimentary canal through which the food passes after it has left the mouth. Above and behind the palate it communicates with the back of the nose, while below it is continuous with the gullet. As we shall see later, it forms also a part of the air passages, since when we breathe the air passes through it on its way from the nose to the lungs. Its walls are composed of voluntary muscular tissue.

We shall presently see that the air tube or windpipe lies in front of the food passage in the neck. The orifice leading into the air tube is closed during the act of swallowing food by a complicated muscular movement, which converts it into a narrow chink, and so prevents the food from passing into the air passage. In front of the entrance to the air passage (glottis) there is a little leaf-like flap of cartilage covered by mucous membrane which is called the epiglottis. This projects upwards immediately behind the base of the tongue. In marsupial animals (kangaroos) this is an important structure, but it has no function in man.

*The Gullet* or *Œsophagus* is the name of the muscular tube which conveys the food from the pharynx to the stomach. It passes through the lower part of the neck into the thorax, where it lies beside the bodies of the vertebræ. The muscular fibres in its walls are entirely of the involuntary kind. After piercing the diaphragm it ends in

*The Stomach*, which is merely a peculiarly shaped dilatation or widening of this particular part of the ailmentary canal. In the mucous membrane of the stomach there are some special glands whose secretion contains a special ferment called pepsin. These glands are therefore called the peptic glands. The walls of the stomach contain a large amount of involuntary muscular fibres, which are arranged in layers. The region where the lower end of the stomach becomes continuous with the small intestine is called the pylorus, and here the tube can be closed by the contraction of a strong ring of muscular fibres. The stomach lies immediately beneath the diaphragm, and occupies, not the centre of the abdominal cavity, but the upper left-hand corner.

The stomach is the first part of the alimentary canal which has a peritoneal covering. All that part of the tube which lies above the diaphragm is without peritoneum. The peritoneum exists only within the abdominal cavity.

Continuous with the stomach is the small intestine. This commences at the pylorus, and terminates in the region of the right groin by joining with the colon or large intestine. The small intestine is the longest portion of the alimentary canal, and is about 21 feet long. It lies in numerous coils in the central portion of the abdominal cavity. Throughout its whole extent digestive juices are poured into it from the numerous minute glands of its mucous membrane. The mucous membrane of this part of the gut is peculiar in that it is studded with an enormous number of minute finger-like projections called villi. Each villus contains blood-vessels and the commencement of a lacteal or lymphatic vessel, and it is through them that the chief absorption of the food takes place.

The first nine or ten inches of the small intestine—that is to say, that part which immediately follows the stomach—is called the duodenum, and into this portion are received the secretions of two large and important glands—namely, the liver and the pancreas. Their secretions are conveyed into the gut by means of ducts or channels, which, however, unite before entering the duodenum, so that the secretions of the two glands may be mixed by the time they reach the intestine.

*The Liver* is a very large gland, the largest in the whole body. It lies in the upper right-hand corner of the abdominal cavity immediately under the diaphragm. It is supplied with arterial blood by a large branch from the aorta, but, as we have already seen, it also receives all the blood which is gathered up from the stomach and intestines by the portal vein—that is to say, the blood which contains all the newly-digested food. In the portal capillaries this blood is brought into close relation with the liver cells, with the result that it undergoes some alteration; for these cells manufacture from it (1) glycogen, which is stored up in the liver for future use as a food, and of which we need not further inquire here; (2) bile, which is the name of the secretion of the liver, and which passes along the bile duct into the duodenum.

*The Pancreas* or *Sweetbread*, although much smaller than the liver, is still a large gland. It lies in a horizontal position across the back and upper part of the abdominal cavity, behind the stomach and duodenum. As we have seen, its secretion enters the duodenum with the bile. With the characters and functions of these two important secretions we shall have to deal when speaking of the processes of digestion.

*The Large Intestine* or *Colon* is so called, not because of its length, for it is much shorter than the small intestine, but because it is a much wider tube. It commences in the region of the right groin, and passes upwards on the right side of the abdominal cavity towards the liver. It then passes across the abdominal cavity and down the left side, and ends in the region of the left groin, where it enters the pelvis, by becoming the rectum. To the first part of this portion of the bowel there is attached a little worm-like hollow appendage a few inches long. This is what is called the appendix, and when it becomes inflamed it produces the condition known as appendicitis.

*The Rectum* is the last portion of the bowel. At its lower end it terminates in an orifice called the anus, which is surrounded by a strong circular muscle called the sphincter of the anus.

*The Physiology of Digestion.*—As soon as the food is received into the mouth it is crushed and torn to pieces between the teeth by the grinding action of the lower jaw. At the same time it is thoroughly mixed with the saliva. The salivary glands are stimulated by the presence of the food in the mouth, and during the act of mastication pour out an abundant amount of their secretion. Even the sight or smell of food, especially if it be of a savoury kind, will excite this secretion. This is so well known a fact that it is a common expression to say that any delicious morsel makes one's mouth water. The food is thus reduced to a soft pappy condition, and those parts of it which are readily soluble in water are dissolved. But as soon as the food comes into contact with the saliva, certain portions of it are at once attacked by that secretion, and through its agency undergo a chemical change. There is in the saliva a ferment (ptyalin) which is capable of converting the starchy foods, such as wheaten flour, etc. (which are insoluble) into a form of sugar (which is, of course, soluble).

This, then, is the main function or use of the salivary secretion—to convert the starches into sugar.

The food should be retained in the mouth for a considerable time in order that its mastication may be complete, for this is a very important part of the process of digestion, since the more finely the food is ground up by the teeth the more rapidly will it be acted upon by the gastric juice, and if it be swallowed only half masticated, portions of it may remain in the stomach in a semi-digested state and may pass on in this unprepared condition into the intestine, giving rise ultimately to discomfort or diarrhœa.

### The Nurses' Clinic.

### INSPECTION AND CARE OF THE MOUTH.

The care of the mouth forms one of the most important nursing duties, and it cannot be urged too strongly that any neglect on the part of the nurse may not only cause discomfort to the patient, but may also retard recovery and even endanger life. As a matter of routine, every patient who is in possession of teeth ought to have them scrubbed at least once a day, but preferably night and morning. Babies should have their mouths washed out before and after feeding. Care must be taken to include every part and recess of the mouth in this process. Particles of food are very liable to settle between the gum and cheek, where they decompose and cause a bad taste, loss of appetite, or worse—septic pneumonia. A piece of clean soft linen, dipped in cold water, wrapped round the nurse's finger, does very well, and can be burnt after use. Patients who are disinclined for their food will often take it better if their mouth is thus freshened up before eating, and it is certainly most necessary to clean or let them rinse out their mouth after food, so as to leave no trace of it.

A daily inspection of the mouth forms a part of a nurse's routine work, which generally starts with the taking of the temperature. Now, a thing which has often struck me most forcibly is the little care which is bestowed upon the disinfection of the clinical thermometer. In a large hospital ward there may be six thermometers. Temperatures have to be taken at 6 A.M., and this is generally done with all possible speed, so as to get everything "straight" before the day nurses come on. The result is that as the thermometer leaves one patient's mouth it is shaken down, hurriedly dipped into some antiseptic solution, wiped, and then inserted between the next pair of lips. This is, to say the least of it, very unappetising for the patient, and also obviously most risky. When temperature taking is completed the thermometers are generally placed vertically into a glass vase, containing a piece of absorbent wool and some antiseptic lotion which only half covers them. In the casualty and out-patient departments no more care is as a rule taken with these instruments, and here the danger of serious infection is very much greater than in the wards where at least the case may have been diagnosed, and the so-called infectious patients, including those suffering from tubercle, are supplied with their own special clinical outfit. Now, I suggest that instead of the six thermometers each large ward should possess twice six thermometers, and that while one set is being used, the other six should repose bodily—that is, horizontally—in a strong antiseptic solution, like lotio. acid. carbol. 1—20, or lotio. hydrarg. perchlor. 1—1000. This would give each set about five minutes to get rid of germs, and if in addition they are rinsed in running water before and after insertion we may be fairly certain of not poisoning patients either with germs or antiseptics. At out-patients' and in the casualty department an equally plentiful supply of thermometers should be kept, and here the precaution should be taken of placing each thermometer into a separate bowl, thus allowing a proper system of rotation being carried out. These methods may seem to some extravagant, but I maintain that economy must not be practised at the expense of safety.

After having taken the temperature, pulse, and respiration, and before giving in her night report, the nurse inspects the patient's tongue and mouth. The old saying that the tongue is the mirror of the digestive work of the body still holds good, and all care must be exercised to notice any change in its appearance. Everybody knows what a healthy tongue looks like. There are many departures from this normal condition. The one which most closely resembles it is the dark-red, moist tongue of the so-called "acid dyspepsia," whereas in "alkaline dyspepsia" we have the thick white fur, so difficult to remove and so quickly regenerated.

In all febrile diseases a more or less furred tongue, ranging from a simple white coating to the brown cracked appearance found in the severe forms of enteric fever, is met with. In bad cases of pneumonia the tongue may closely resemble the enteric tongue, and particles of viscid-tenacious mucous will be detected adhering to the various parts of the mouth. The tongue of scarlatina is typical; when the first fur has been thrown off it leaves the tongue diffusely red, the swollen papillæ forming the typical strawberry excrescences. At the onset of measles the presence of Koplik's spots—red patches on the inner surface of the cheek and on the gums—help diagnosis, while in very bad, and specially in neglected, under-fed cases, another symptom ought to attract attention at once; this is a hard red spot on the lips or on the cheek, often the precursor of that frightful scourge—cancrum oris. Within a few hours this spot may change from red to black, spreading with alarming rapidity, while the intense fœtor of the breath leaves no doubt as to the diagnosis.

In whooping-cough the typical white ulcer is sometimes seen under the tip of the tongue. In scurvy the spongy offensive gums, which bleed with the slightest injury, is found. The teeth are loose, and may fall out. A blue line on the gums may be due to lead-poisoning. Any deviation

### THE NURSES' CLINIC—*Continued.*

from the healthy "sweet" odour of the breath must attract our attention. A nurse must also be careful to notice if there is any and what departure from the middle line when the tongue is put out, and any difficulty in protruding it. Any swelling, either of the soft or hard parts of the mouth, should also not escape inspection. With regard to the tongue of infants, it will have been noticed that it is often spread with a white fur after suckling. Older children's tongues may present a peculiar mapped appearance, the dorsum of the tongue exhibiting various greyish-white figures, which contrast markedly with the red colour of the normal areas.

White patches on the tongue and mucous membrane of the mouth and palate are often seen, and in newborn babies adhesions of the tongue, the so-called tied tongue, may be found in very rare instances. A cleft palate is too obvious to require mentioning.

All these deviations from normal should be carefully noted and reported. In addition the state of the teeth must be watched. Carious teeth and old stumps are often the source of serious digestive trouble; while swollen gums in babies, if detected, will often explain a dark, hitherto undiagnosed condition.

Characteristic changes occur in syphilitic teeth, especially in the permanent upper central incisors, which may be markedly dwarfed, notched, and peg-shaped, the other incisors being so to a lesser extent.

Rocky, rugged teeth, studded with pits like a thimble, must be distinguished from the above, and may be the result of mercury treatment, malnutrition in infancy, and many other causes. Teeth are quickly discoloured and affected by iron, hence it is advisable that fluid iron medicines should be taken through a glass tube and the mouth well washed out after each dose. I would also strongly urge that a daily thorough inspection of every patient's throat be made in children's hospitals. In this way many a tiresome and possibly fatal spread of infection may be avoided.

It is necessary to add that a marked alteration of colour in the tongue may not as a matter of course denote disease. Some foods, and drugs like iron, may dye the tongue a rich blue or black without causing any damage.

As to the treatment of abnormal mouths, it must be left to the medical man. Without his leave nurses are only allowed water, and this is used with the greatest liberality. Where the toothbrush is not sufficient, soft clean rags, or a small spongeholder and absorbent wool swabs, wrung out of water or the mouth-wash prescribed, will probably do all that is wanted, only it must be done with great thoroughness and gentleness, missing no fold or crevice. An enteric tongue will require a good deal of gentle patient scouring, and the removal of the viscid expectoration of pneumonia calls for unremitting care. In some cases the tongue will have to be depressed to reach the roof of the mouth, but a nurse should not be harsh, or she will frighten the patient and may make him sick.

Sore, cracked lips may require prescribing for, but a nurse should remember that she must first well cleanse and dry the part, so as to give the application a chance of reaching the disease and producing a cure.

### Incidents in a Nurse's Life.

Contributions to this column are invited.

### THE TOMKINS' FAMILY.

"Ben Tomkins, pot-hawker, No. 7 Lime Street, is suffering from pneumonia. Will the nurse kindly call and make him comfortable?"

The above request was telephoned to us one morning by a local doctor.

Lime Street is in my district. As soon as possible I found my way to No. 7, and knocked at the door.

It was opened by a rough, pleasant-looking woman of about forty-six or forty-eight years of age.

"Good morning. Does Mr. Ben Tomkins live here?"

"Yes, 'e does, nurse. Come in. I'm right glad ter see yer, for I'm about at far end wi' mysel'. It's my 'usband's father as is bad; 'e's been laid up this three days, an' now Miranda, my married daughter ('er an' 'er 'usband lives 'ere along wi' us; 'e's in t' pot line) 'as been an' 'ad a baby durin' last night. If it 'ad n't been fer t' neighbours poppin' in I don't know what I should ha' done; because there is my 'usband an' three lads in family as well."

I sympathised with her.

On proceeding upstairs I found Mr. Ben Tomkins very poorly; his temperature was 104° and the pulse very quick.

During the three days he had had practically no nursing. His daughter-in-law and the neighbours had given him drinks, but nothing else was done for him.

I washed and made him comfortable, and instructed "young Mrs. Tomkins" how to go on until I came again in the evening.

"God bless yer, nurse; may yer never die," the old man murmured as I bade him "Good morning." In the evening he was a shade better.

The following morning found him better still, but Mrs. Tomkins was worried about her daughter's baby.

"Do you mind just lookin' at Miranda's baby, nurse? It's been throwin' up summat awful."

"What has it had?" I asked.

"It's 'ad nuthin' but a few spoonsful o' gruel, made wi' good milk."

"But gruel is not suitable food for so young a baby; and they need so little of anything at first."

"Well, nurse," she said humouredly, "times was an' times is; but if you'll believe me, when I was 'avin' my childer, the first basin o' gruel as was brought me after they was born I allas gave t' baby it's share ont."

I experienced no difficulty in believing her.

"How many children have you had, Mrs. Tomkins?"

"Ten, nurse," she answered proudly.

"And how many living?"

"Four, nurse; t'others all died when they was a few months old."

"Perhaps," I said, smilingly, "the other six might have lived if you had not given them gruel when they were so young."

"Oh no, nurse," she laughed incredulously. "I can't ha' that. It were t' will o' the Lord. Why it says in t' Bible as 'the Lord gave an' the Lord taketh away.'"

Eventually Old Ben recovered, much to the doctor's pleasure and surprise, as his age was sixty-six; but he was wonderfully wiry, and too, he had always spent the greater part of his life in the open air, hawking pots.

A few days ago he brought to the "Home" two pudding basins, three stew jars, and a brown tea-pot, with the request that they should be given "ter t' nurse fer our distric', with Ben Tomkins' respecs, an' 'e 'opes she'll 'ave 'er 'ealth ter use 'em."

### The Jubilee of the London Biblewomen and Nurses Mission.

The first public meeting in commemoration of the Jubilee of the London Biblewomen and Nurses Mission being announced to take place on the 18th of this month, the developments of the Institution are exciting special interest. As it is well known, the Biblewomen started in 1857 under the auspices of Mrs. Ranyard, and in 1868 the need of the suffering for physical as well as for spiritual help so impressed the Founder that a few nurses, trained as much as was then possible, were engaged for house-to-house visitation, on similar lines to those of the East London Nursing Association, which had just been organised. Though now, of course, the preparatory training which the workers receive is very different

The Parker Street Hostel.

from what it was at the inauguration of the scheme, the ideal then conceived is still carried out—namely, "the employment of women possessing a trained efficiency united to a sense of Christian vocation."

### The Training.

Candidates now desiring admission naturally divide themselves into two classes, those wishing for training—who should not be under twenty-five years of age—and those who have already completed their course of study and desire employment. Both classes are required to reside for a short time in the Hostel and Training Home at Parker Street, Holborn, to test their suitability for the position. Here, in addition to the daily visiting with an experienced worker, the candidate is given a three-fold course of study embracing systematic Scripture instruction, tuition in the principles of thoughtful relief-giving, and lessons on hygiene, so as to prepare her for her work as a health missioner. Untrained candidates, before being sent to the general wards of one of the large London hospitals for two years, are asked to sign an agreement, undertaking to work for the Society for three years from the time that their training is completed. At the end of the two years the nurse returns to the Training Home to receive such instruction as she needs in district work, before she takes up her residence in her appointed parish or district. During the first two years of district work she has to qualify herself for the examinations held by the Mission, for which upon completion of her engagement she is given a certificate, should her work and conduct prove satisfactory. Candidates who apply ready trained—and these are becoming increasingly numerous—are not required to sign any agreement, the engagement

A Group of Nurses and Biblewomen.

being terminable at any time by a month's notice on either side, unless the nurse receives maternity training at the expense of the Mission, in which case she is required to bind herself for one year in return.

### Work of the Nurses.

When the nurse is appointed to her own district she is placed under the care of an experienced Superintending Sister who visits her regularly, directs her medical reading, prepares her for her final examinations, and may be said to correspond to the Sister in a hospital ward. The working day usually consists of seven hours, and begins at 10 a.m. Nurses are not allowed to act as midwives, nor to attend infectious cases unless specially set apart for the purpose. Night work is not undertaken unless in extraordinary circumstances. The uniform is obligatory, consisting of a black cloak and bonnet when in the streets, and grey-blue print dresses and linen aprons when at work. Whether indoors or out, it is necessary for every nurse to wear the badge of the Mission, which is a bronze cross having upon it the words "Laborare est Orare" and "Orare est Laborare," the date 1868, and the initials of the Founder, L. N. R. These badges, hung from a narrow red ribbon, serve to distinguish the nurses of the Society. Each nurse is given a case-book and a bag, furnished for her by the Mission, which contains everything she is likely to require of an up-to-date pattern. In addition to medical stores, needful instruments, a mackintosh, and a flannel apron, the kit includes a dressing-mat. This is highly glazed on both sides, is easily cleansed, and upon it the nurse lays her dressings, so that in a dirty house they may be protected. She is also given for her loan cupboard, blankets, sheets, bed jackets, night-gowns, and many articles which may occasionally seem indispensable for the successful nursing of a patient. Every month the nurse attends at the head office of the Mission in Adelphi Terrace, Strand, and replenishes her bag, having previously sent in a list of her requirements so that they may all be in readiness for her. The nurses work entirely under the medical men of the neighbourhood. They receive no fees from the persons they attend, but grateful patients are encouraged to send thankofferings to the General Fund.

### The Convalescent Home.

Twenty years ago a home for convalescent patients was started and since that date 7,000 guests have been received. The results have been eminently satisfactory. Patients who have recovered under the care of the Mission nurses, but who appear to need further rest and care are sent to St. Leonards-on-Sea, where, owing to the constant supervision of the matron (a trained nurse), any treatment prescribed can be carried on, and good results in consequence are far more rapid than in some of the Convalescent Homes where no nursing can be obtained.

### How the Mission Helps the Nurses.

The Mission is particularly careful to look after the welfare of its nurses in their work, and is interested in their future. A Sister goes round regularly and visits the nurses so that they need never feel themselves thrown entirely upon their own resources, but in times of pressure can always obtain any assistance wanted. Lectures by well-known doctors are delivered to the nurses occasionally at the Hostel, and the Assistant-Matron and Sisters help in introducing new methods and circulating information. Five weeks' holiday is given, the first week of which may, if desired, be spent in the Home of Rest for Workers near Brighton, the remaining weeks there or elsewhere. Here, also, an ailing nurse can soon be strengthened and refreshed to return to work. Last of all, the Council of the Mission has federated with the Royal National Pension Fund in order to assist any nurses under forty who wish to insure in the Fund. The Mission will take out a policy, on the returnable scale, for a pension of £11 5s. a year to be payable at fifty-five years of age, if the nurse will, on her own account, take out one of not less than £7 10s. per annum. As long as the nurse remains in the service of the mission the £11 5s. is paid, but if after five years the nurse wishes to leave, the Mission policy may be assigned to her for her to continue, or to realise, as she sees fit.

### Public Recognition of the Work.

For the first time the Hospital Sunday Fund has decided this year to extend to nursing institutions its benefits, and one of the three institutions selected is the Biblewomen and Nurses Mission. This fact must be a matter of congratulation to Miss Andrews, who, as Honorary Superintendent, succeeded Mrs. Selfe Leonard's able administration. There are now sixty-five nurses in the employ of the Mission, who, although they receive fair salaries, are chosen mainly because they regard their labours from the highest standpoint.

### Central Midwives Board.

### ISSUE OF AN IMPORTANT MEMORANDUM.

A meeting of the Central Midwives Board was held on Thursday last week. There were present Dr. Champneys (Chairman), Mr. Ward Cousins, Dr. Dakin, Mrs. Latter, Miss R. Paget, Sir William Sinclair, Miss Wilson, and Mr. Parker Young.

The first business considered was a letter from the Clerk of the Privy Council enclosing copy of a resolution passed by the Board of the National Maternity Hospital, Dublin, in favour of examinations being held by the Board in Dublin, together with another letter from the Secretary of the Rotunda Hospital, Dublin, to the same effect. Similar letters from other authorities had been considered at an earlier meeting by the Board, and they had felt that as Ireland had expressly desired to be untouched by the Act, the Board could not justly incur expense on its behalf. In view of the letter from the Privy Council, favouring the suggestion, the Board decided to refer the question to the Standing Committee for further consideration, though the general feeling seemed to be that the idea was not very feasible.

A question arose on the letter of a medical officer of health as to whether a certified midwife was obliged to keep a register of those cases which she attended only in the capacity of a monthly nurse under a doctor, and it was decided that this was in no way her duty, the Board pointing out that in cases of doubt application could always be made to the doctor alleged to be in attendance.

A memorandum from the committee on applications as training schools from Poor-law institutions was accepted. The memorandum distinguishes between the two classes of institutions, workhouses and workhouse infirmaries, pointing out that in the former the medical officer is not usually resident, the general discipline of the house is in the hands of untrained and non-medical management, and the nurses are not generally fully trained, while in the latter the sick and lying-in cases are under one or more resident medical officers, a trained matron, and fully trained nurse and the needs of the sick are the first care of the officials. The memorandum goes on to point out that if an institution is recognised by the Board it is able to obtain a better class of nurse, and that the knowledge that free training in midwifery may be obtained at the end of three years' general training attracts many superior women who cannot afford to pay fees. The Board must consider each case on its merits, and the institution must be inspected by an inspector accustomed to the work, local prejudices and feeling running too high to make local inspection truly valuable and independent. Reference is made to the Board's previous decision that no such institution shall be approved as a school in which 75 deliveries do not take place annually, and that inspection is absolutely necessary. With regard to the approval of doctors desiring to teach pupils in Poor-law institutions which are too small to be approved schools, the memorandum suggests that in future as a rule a number of not less than 60 cases annually shall be essential for such approval.

Sir William Sinclair dissented strongly from the memorandum, and said that it was untrue to say that superior women went to the workhouse infirmaries, and that he certainly would not employ any such woman in his private work; and as to local inspection, the medical officer of health was quite independent and could give a true report. Miss Paget and Miss Wilson spoke very warmly on behalf of workhouse infirmary nurses.

Mr. Parker Young moved that in the event of a midwife's certificate being lost, injured, or destroyed, a statutory declaration to that effect be required, together with the payment of 2s. 6d., should a new one be requested. It was decided that in no case should a second certificate be issued, but only a voucher of the previous issue of a certificate.

The date of the next meeting was fixed for June 28.

### Canon Scott-Holland on Nurses.

Nurses and friends of all district nursing societies received an invitation to attend in the crypt of St. Paul's on Tuesday last to hear a special address by Canon Scott-Holland. The seating accommodation in the crypt proved insufficient for the numbers who availed themselves of the opportunity, and the vergers were busily occupied in bringing further relays of chairs.

Canon Scott-Holland took for his text Psalm civ. 15, "Oil to make him a cheerful countenance," and said that what had struck him most about nurses ever since he came in contact with them was their cheerfulness. There was no class on whose faces one was so sure to see confidence and gaiety as nurses. Whence did this cheerfulness come? They were in the midst of misery; they were where the shadows had fallen; they knew the bitter story of men and women and all infamy and degradation and horror. They knew often the uselessness of service, that their pains were often in vain, that they could not save nor heal, and yet they always carried with them that look of good cheer and confidence. Others, outsiders, if they came into ever so slight touch with these miseries became oppressed with darkness and despair. There was a kind of optimism that provoked, because it refused to recognise facts, or said there were no such things as pain and suffering. But the optimism of the nurses disguised nothing and yet they came out of the darkness with news of good cheer. Now, where did they get this cheerfulness from? First there was the oil of activity: the need to be active in face of facts. It was a splendid thing always to know what to do; nothing mattered as long as they could do something. Next there was the oil of efficiency: doing something well; the joy of an artist and craftsman. Then there was the oil of tenderness and sympathy: always moving about bringing something of relief, some ease to each poor sufferer; teaching them there was still somewhere in the world some love and some care for them. There was the oil of vocation: "Here am I; send me." And then there was the oil of healing. Christianity found a great parallel in the art of healing. Christ when on earth spent His time in healing those terrible diseases of olden times. Lastly, there was the oil of hope: they asked nothing as to the past, but lived for the future. If they would keep themselves in a cheerful countenance they must gain power to do so from the Eucharist, the great thanksgiving. They had lately lost one who had done so much for them, and when they thought of her they thought also of all those who had served and helped in their day.

### Nurses' Missionary League.

### ANNUAL MEETING.

The fourth annual meeting of the Nurses' Missionary League was held at University Hall, Gordon Square, on May 30, beginning at 9.30 A.M. After Miss Van Sommer had spoken of the message that only a Christian woman can carry to the women of the non-Christian world, Dr. Wilhelmina Eger gave a graphic account of the beginning and development of the medical work at Multan. Multan is a town in the South Punjaub, with a population of 70,000, standing in a district comprising over 6,000 square miles and upwards of a million inhabitants, for whom there was, twenty years ago, no qualified doctor, midwife, or trained nurse to minister to women in times of sickness. The Female Education Society was the first to send out a lady missionary, but she failed to secure any opening. Later Miss Wadsworth and Dr. Eger were sent, and, after nine months, a little mud house with a yard behind was secured and a dispensary and out-patient department were started. For years Dr. Eger worked almost alone with the help of an old woman: all the dusting and cleaning, dispensing and teaching, visiting in the city, and every variety of work was entirely dependent on her time and strength, and the work was closed when Dr. Eger came home for her first furlough. After fifteen years a hospital was built on a site outside the town, and now, after five years, the number of in-patients has risen to 462, and there is still accommodation in the spacious buildings for as many more. As an illustration of how prejudice is overcome, Dr. Eger told of an operation performed on a little girl kept in strict purdah because she was a descendant of a saint, by whose shrine, the famous Pir Shrine, her family lived; after much difficulty the family were persuaded to let the child be carried to the hospital for the necessary operation, and its success did much to establish confidence in Dr. Eger's work.

There is immediate need of a fully trained Christian nurse to be matron, sister, staff nurse, all in one, to train the band of native nurses whom Dr. Eger has already on the staff.

### Everybody's Opinion.

[Correspondence on all subjects is invited, but we cannot in any way be responsible for the opinions expressed by our correspondents. No communication can be entertained if the name and address of the correspondent are not given as a guarantee of good faith, but not necessarily for publication. All correspondents should write on one side of the paper only.]

### POOR-LAW NURSES AND SUPERANNUATION.

[With reference to the correspondence and notes which have appeared in The Hospital *Nursing Mirror* on the above subject, we have received the following letter, dated May 25, from the Local Government Board. It will be seen that the Local Government Board feel, as we consider rightly, that each case of the kind must stand upon its own merits. All nurses, therefore, who are promoted to a higher office, and who may consider themselves in consequence eligible for a pension, should take steps to have their position exactly defined under the Acts regulating the pensions of poor-law officials.—Ed. The Hospital.]

I am directed by the Local Government Board to advert to your letter of the 12th instant, relative to the position of an officer who has contracted as a nurse out of the provisions of the Poor-law Officers' Superannuation Act, 1896.

In reply I am directed to state that the questions involved are not such as would be the subject of general determination by any Order which the Board are empowered to issue and which would necessarily be binding upon Poor-law authorities in their administration of the Poor-law Officers' Superannuation Acts.

Any decision which the Board might give under Section 18 of the Act of 1896 would have regard and be limited to the actual facts of the particular case brought before them under that section. I am, Sir,

Your obedient servant

John Lilluby.

Assistant Secretary.

### BABIES' NURSES.

"Noblesse Oblige" writes: I think that a few words of thanks and apreciation are due to "Sarah Gamp" for her admirable words of sound, practical common sense. Such women as she are those who prove themselves to be the successes of their profession—the real ladies (irrespective of any sphere they may have been born in) who by lives of devotion to their work keep up the standard of their profession, and, what is of the greatest value, imprint upon the world the desire to follow their good example and to go on and do likewise. Instead of tarnishing the highest of ideals with the verdigris of controversy and idle words, they increase their brightness with the inspiration of a noble life. Personal experience teaches me that the nurse who talks loudest of the rights and wrongs of her profession —of "Nursery Maids and Uniforms," "of her birth-right and position as a lady"—is the one above all who has least occasion for so doing, and is also the one who is the most likely to prove a failure in her profession. The nurses who are the heart and life of their profession, and of whom I, even as a head, am proud to say I look up to, honour, and love, are those from whom I never hear a word upon any of these controversial subjects; their work is too real, too absorbing for them to waste time and words upon superficial trivialities and the idle battle of tongues.

### SARAH GAMP AND THE MODERN NURSE.

"Dudley L. Beaumont" writes from 104 Church Road, Upper Norwood: In reference to your very interesting report of Miss Genn's "onslaught" on trained nurses, may I, as one who has seen a great deal of them during the last six years, say that I think that the address is very bitter, and unfair to lady nurses, but that I quite agree with the lady who said that she would rather have someone to nurse her who would obey her orders, instead of one quoting and carrying out the doctor's wishes." I do not think that the lady nurses are coarse, or that they always "set their caps" at the students, though I have known one very modest nurse who married very happily the manager of a theatre. I have met with "the pert, cocksure person" who is a hindrance to her patient and her employer, but, fortunately, she is not by any means universal. I have noticed that both good and bad nurses alike think far more of keeping their places than of their patient's comfort, and study more to please the doctor than to minister to their patients' welfare.

"A Non-wearer of Creaking Shoes" writes: I was glad to read your report concerning Miss Genn's address. I think that every private nurse, of whom I am one, will feel grossly insulted. Since I have been a private nurse I have met many high-minded, self-denying women, who have been true followers of Miss Nightingale, and whom I have hoped to copy, but never can be as good; for them and not for myself I feel how unjust her remarks are. Concerning nurses marrying doctors, they do so, and usually are "the better half"; but I doubt if Miss Genn can bring forward one case in which a man has been "professionally ruined" through being accepted by a nurse. In our profession we wait to be asked by the gentleman. Many widowers do marry nurses, but I think that is in their favour, and shows that they paid adequate attention to their patient. Many nurses go again and again to their patients and their friends, and even get asked to visit for holidays; if they were what Miss Genn represents, they would not be tolerated. Mrs. Gamp was in some cases a capable and sensible woman, but I do not intend comparing trained nurses with her. We judge of vaccination by the results, and so with trained nurses. If a nurse has time for fancy work, and does not neglect her patient, why should she not pass her spare time away in doing such work—work for charity bazaars? None of us want our patients to buy our work; we can always dispose of it by sale privately if we wish to do so. Miss Genn does not mention that sometimes trained nurses go to houses where the residents cannot afford a servant. Who then keeps the room clean and does the cooking for her patient but the nurse? I do not think that we are wanting in time of need. Miss Genn has evidently made a mistake about a nurse's fees. I know that in the North of England there is a great need for nurses to form a union to prevent fees being cut down.

### Where to Go.

New Hospital for Women, 144 Euston Road, Thursday, June 14, 2.30 to 7 p.m.—Sale of children's clothing, toys, dolls, books, and other articles. There is a stall in charge of the nursing staff.

### Appointments.

[No charge is made for announcements under this head, and we are always glad to receive and publish appointments. The information, to insure accuracy, should be sent from the nurses themselves, and we cannot undertake to correct official announcements which may happen to be inaccurate. It is essential that in all cases the school of training should be given.]

Bradford Eye and Ear Hospital.—Miss Jessie Elms has been appointed sister. She was trained at the Hahnemann Hospital, Liverpool, and has since been sister at the Eye and Ear Infirmary, Liverpool, the Central London Ophthalmic Hospital, and the Birmingham and Midland Eye Hospital.

Cumberland Infirmary, Carlisle.—Miss Beatrice Stevens has been appointed sister. She was trained at the Wolverhampton and Staffordshire General Hospital, where she afterwards acted as holiday sister. She has since been sister and night superintendent at the Salop Infirmary, Shrewsbury.

Holborn Union, Mitcham.—Miss Selina Mary Nash has been appointed superintendent nurse. She was trained at Southwark Infirmary, East Dulwich, London, where she subsequently became staff nurse and sister. She has since been sister at the Hospital Convalescent Home, Parkwood, Swanley, and has done private nursing.

Nursing Institute, Bexhill.—Miss H. R. Whealler has been appointed matron. She was trained at the Coventry and Warwickshire Hospital. She has since held the post of charge nurse at the Windsor Royal Infirmary. She has also done district work as Queen's nurse.

Royal United Hospital, Bath.—Miss Katherine Grice has been appointed staff nurse. She was trained at the Royal Southern Hospital, Liverpool, having previously been assistant nurse at the Royal Hospital for Incurables. She has since been attached to the Royal Southern Hospital, Liverpool.

St. Anne's Home, Herne Bay, Kent.—Miss Florence Kite has been appointed head nurse. She was trained at Greenwich Infirmary, where she was afterwards staff nurse. She has since been ward nurse at Camberwell Infirmary, superintendent nurse at Canterbury Infirmary, superintendent nurse at Ladywell Workhouse, Ladywell, S.E., superintendent nurse at Trowbridge and Melksham Infirmary, and charge nurse at St. Anne's Home, Herne Bay.

Salop Infirmary, Shrewsbury.—Miss Mary L. Appleyard has been appointed matron. She was trained at St. Bartholomew's Hospital, London, where she afterwards became ward sister and assistant housekeeper. She has since been night superintendent and assistant matron at the Royal Hospital for Sick Children, Edinburgh.

Solihull Union Workhouse.—Miss Elsie Price has been appointed Superintendent. She was trained at Liverpool Workhouse Infirmary and has since been nurse at Worcester Union Infirmary, and at King's Norton Union Infirmary.

### Presentations.

Bradford Eye and Ear Hospital.—Miss Jeannie Brooke, on leaving the Bradford Eye and Ear Hospital where she has held the post of sister for the last 3½ years, to take up her duties as matron of the Bierley Hall Hospital, Bradford, was last week presented with a silver tea service from the Committee and medical staff, half a dozen silver tea-spoons from the matron, an antique copper tea-kettle on stand from the nurses, and photograph frames from the domestic staff, besides some personal gifts.

### Death in our Ranks.

We regret to hear of the death of Miss Mary Hay, a member of the Army Nursing Service Reserve. She worked for five years in South Africa, only returning to this country last summer for a well-earned rest from her arduous duties. Thinking that her native air in Scotland was all that was required to re-establish her health, she was taken there, but gradually became worse, and passed peacefully away on May 13, after several months of intense suffering, patiently borne. She was nursed until the end by her devoted sister.

### Novelties for Nurses.

(By our Shopping Correspondent.)

### A NEW DIFFUSER.

Messrs. E. and R. Garrould, of 150 to 160 Edgware Road, W., have supplied a distinct want in their new diffuser sponge. Everyone who uses sponges must have been struck by two facts—how expensive they are to buy, and how soon they wear out. This new article consists of small pieces of pure sponge enclosed in a very strong close netting, so loosely arranged that quite a volume of water can be absorbed, and yet the price of the largest size is only 2s., the smaller costing 1s. and 1s. 6d. These diffusers, it is safe to predict, will soon become very popular.

### THE OKTIS CORSET SHIELDS.

Nurses are especially prone to break the side supports of their corsets, as they have to bend and stoop more than most other individuals. The rustless supports form a simple and effectual remedy. They are formed of four steels or of rustless zairoid, covered with sateen and connected with a strap of the same material in the middle. The steels are placed far apart, so as to give elasticity, and they can be sewn to the corsets in a few minutes. The Zarna busk-protector is another useful invention to save the busk from destruction. Both these articles are obtainable of most drapers.

### HOBART CYCLES.

Every year new cycles are introduced, but I know of nothing superior to the "Royal Hobart," with two-speed gear and oil-bath case. The advantage of the latter is that the gear is always working under the best and easiest conditions; consequently the machine will last a very long time. It may be mentioned that nurses desiring to purchase this machine can buy it direct from the makers, Hobart Bird, Limited, Coventry, without the intervention of agents, thereby saving a considerable sum of money. Numerous testimonials include one from a purchaser in Wales who has ridden 12,000 miles on one of these, and states that it shows no signs of wear; and another, after two years' constant use, says that the machine is in first-class condition, and he has never spent a penny upon repairs.

### TRAVEL NOTES AND QUERIES.

By our Travel Correspondent.

Notice.—Those correspondents who are taking their holidays in August will not perhaps see answers to their questions at once in this column, because, owing to pressure on our space, I must attend to the wants of the early holiday-makers first. But no one will be forgotten.

France for a Week (Old Maid).—I can arrange exactly what you want, and you can keep within the smaller sum you name. Take the Friday boat from Southampton to St. Malo (addresses will come by post); stay there till Saturday, leave by early train for Mont St. Michel, going *via* Dol. You will have some time to wait there. Go into the town and visit the pig market, which is one of the curious sights of Brittany. From Dol to Ponterson by train, and there you will find omnibuses going to the Mount. On arrival go to Hôtel Poulard Aîné; be sure about the *aîné* as there are two hotels of the same name. Terms 7½ francs per day. Leave Monday morning for St. Malo. Stay there till Friday or Monday, according to the time at your disposal, and return to England. Second-class return fare to St. Malo from London, £1 19s. 10d. Boats run both ways on Mondays, Wednesdays, and Fridays only.

Addresses Recommended (L. K.).—Many thanks, they will be valuable.

Wiesbaden for two Friends (Water cure).—Your route will be Harwich—the Hook and Cologne. Hôtel du Nord, Wilhelmstrasse, has terms from 5 marks, and I think has a covered way to the Baths; Hôtel Grünes Wald, 10 Markt'strasse, 4 to 6 marks; Hötel Tannhäuser, 8 Bahnhofstrasse, from 4 marks. The fact that two people occupy one room makes no difference as to terms, so you may as well have the additional comfort of separate ones.

Apartments in Malvern (A. B.).—Thanks for letter. I shall be very glad to give your address to anyone applying to me.

Addresses in Paris (R.).—I do not think you can do better than to go to Hôtel Britannique, Avenue Victoria; terms 7½ francs. It is central, and the people most obliging. Should it be full up, try Hôtel de Londres et de Milan, 8 Rue St. Hyacinthe. No, you do not save by taking tickets from Cook, but you are spared some thouble.

Llandudno in June (Jem).—Write to the following addresses, enclosing stamp for reply, and ask their terms. They will, I think, be about what you need: "Milverton House Boarding Establishment," "Heath House Private Hotel, Nevile Crescent," and "Ormes Cliff Boarding Establishment." Also "The Elms" and "the Métropole." So early in the year you would be able to arrange for terms under £2 probably. There are coaches and steamers that make many excursions, about which you will receive information wherever you stay. You can visit the Great Orme, the Little Orme, Conway, with its bay and Castle, and, by sleeping out one night, you can see Llanrwst, Bettws-y-Coed, and Capel Curig.

The Coast of Normandy (Mignon).—I fear the amount you speak of cannot possibly be made to cover the journeys and expenses of two people for three weeks. Normandy is out of the question, being rather expensive. There are only two places I can recommend at your terms, one being the Bay of St. Malo and the other in Belgium. Failing these, would you like Cornwall or Devonshire? Let me hear again.

Holiday in Switzerland.—On the same lines as previously I propose to organise another tour to Switzerland this summer, and shall arrange to visit the Bernese Oberland if a party is formed. We shall start on Tuesday, August 7, and those coming straight back will arrive in London on Tuesday, August 21, the holiday being organised for that fortnight. The cost will be 10 guineas. This includes second-class travelling from, and back to, London (third-class on the mountain railway above Interlaken), and full board (including breakfast, lunch, and dinner), and lodging at comfortable hotels. Expenses connected with luggage must be defrayed by the owner. A few single rooms are available at an extra cost of 12s. The tickets will be available for 25 days for those wishing to extend their stay. The route outwards will be via Dover, Calais, Basle, and Berne. We shall divide our time between Grindelwald and St. Beatenberg, arriving there on Wednesday, August 8. On Tuesday, August 14, the Grindelwald half of the party will go to St. Beatenberg, and the St. Beatenberg party to Grindelwald. The return journey will be commenced on Monday, August 20, and will be via Lake Brienz, the famous Brünig Pass, and Lucerne, with an option of coming back via Paris without extra cost. Those who wish to prolong their stay can easily visit Adelboden, Kandersteg, Wengen, Mürren, or other well-known centres. Application should be made at once, as the party is limited in number and the hotels fill early, to L. Edna Walter, B.Sc. Inspector of the Board of Education, 38 Woodberry Grove, Finsbury Park, London. N.

### Notes and Queries.

### REGULATIONS.

**The Editor is always willing to answer in this column, without any fee, all reasonable questions, as soon as possible.**

**But the following rules must be carefully observed.**

1. **Every communication must be accompanied by the name and address of the writer.**
2. **The question must always bear upon nursing, directly or indirectly.**

**If an answer is required by letter a fee of half-a-crown must be enclosed with the note containing the inquiry.**

*Housekeeper as Matron.*

(119) Do you know of any isolation hospital managed by a housekeeper instead of a matron?—*E. J.*

If you mean an isolation hospital in occupation, we do not know of such a case, and it would be most improper unless a nurse in charge had full control of the nursing department.

*Cottage Homes.*

(120) What are the duties of a matron of Cottage Homes for Children?—*M. H.*

We cannot possibly enumerate them here, but generally the matron would supervise the whole arrangements and see that each Home was properly conducted. When engaged a matron would be informed as to her duties, but she would not be likely to be successful unless she had had similar experience or a great liking for such work.

*Training.*

(121) Please tell me where I can train in a small hospital near London?—*Anxious Reader.*

Procure "How to Become a Nurse" from the Scientific Press, 28 Southampton Street, Strand. You will find all information there.

*Convalescent Home Song.*

(122) Where can I get the Convalescent Home Song? Is it set to music?—*Matron.*

Perhaps some of our readers can help you.

*Children's Nurse.*

(123) Where can I get experience in the nursing of sick children? I am 27.—*A. F.*

Try the Glasgow Royal Hospital for Children.

*Massage.*

(124) Could a certificated Norwegian masseuse get work in England?—*Norwegian.*

Write to the Secretary of the Institute of Trained Masseuses, 12 Buckingham Street, Strand, W.C.

*Home in Gloucester.*

(125) Can you tell me of a Home for training nurses under religious sisters in Gloucester?—*M. F.*

This is probably a private institution, and we cannot give you the address.

*Under Twenty.*

(126) Are there any hospitals which take probationers under 20?—*Mater.*

No general hospitals take probationers so young, but some children's hospitals admit them at 20. Write for "How to Become a Nurse," price 2s. 3d. postage paid, from the Scientific Press, 28 Southampton Street, Strand.

*Homes for Incurables.*

(127) Are there any Homes for Incurables in Lancashire?—*Q. L. J.*

The Manchester Northern Counties Hospital for Incurables.

*Swabs.*

(128) Can you tell me of anything to prevent carbolic acid 1-20 put on sterilised swabs going brown in a few days; also how long does carbolic keep its strength? Is it necessary to boil the swabs again in a week if they are not touched during that time in order to keep them aseptic?—*O. L. C.*

Swabs should be made daily; if it is necessary to keep them for a few days they should be stored in glass ware, as any metallic contact is apt to lead to discoloration. Carbolic acid keeps its strength indefinitely. If the swabs have not been stored in a hermetically sealed glass disk, they should certainly be resterilised before use.

**Handbooks for Nurses.**

| | Post Free. |
|---|---|
| "How to Become a Nurse: How and Where to Train." | 2s. 4d. |
| "Nursing: its Theory and Practice." (Lewis.) ... | 3s. 6d. |
| "Nurses' Pronouncing Dictionary of Medical Terms." | 2s. 6d. |
| "Complete Handbook of Midwifery." (Watson.) ... | 6s. 4d. |
| "Preparation for Operation in Private Houses." ... | 0s. 6d. |

Of all booksellers or of The Scientific Press, Limited, 28 & 29 Southampton Street, Strand, London, W.C.

### For Reading to the Sick.

ON THE ANGELS—(SERVICE).

Still through the cloven skies they come,
With peaceful wings unfurl'd;
And still their heavenly music floats
O'er all the weary world:
Above its sad and lowly plains
They bend on heavenly wing,
And ever o'er its Babel sounds
The blessed angels sing! *Anon.*

The angels must watch with eager interest the man who is going through hard struggle which tries his spirit—they watch to see that he endures. They do not try to make the struggle less hard, but in the moment of faintness or wavering—if there be such a moment—they whisper cheer and encouragement, that the man may not faint. We have a beautiful illustration of this in our Lord's experience in Gethsemane. Angels came—not to take the cup away, but to strengthen Him, that He might not sink down in the darkness. There is a picture by Domenichino which represents the scene on Calvary on the evening after the Saviour's body had been taken down and laid in the grave. The cross is empty. An angel stands beside the crown of thorns which lies there, feeling with the tip of his finger one of its sharp points. His face wears a look of wonder. He is trying to find out the meaning of suffering, but he cannot understand it, nor fathom its depth. . . . The same thought is suggested in the words, "which things the angels desire to look into." Surely it is worth while to give thought and attention to the wonderful things of Christ's redemption, since even the angels find in them mystery worthy of their deepest study.—*Dr. J. R. Miller.*

And he dreamed and behold a ladder set up on the earth, and the top of it reached to heaven; and behold the angels of God ascending and descending on it.—Gen. xxviii. 12.

He had seen in vision a ladder reared against the sky, and angels ascending and descending on it. Exceedingly remarkable. Immediately after his transgression, when leaving his father's home, a banished man, to be a wanderer for many years, this first meeting took place. Fresh from his sin, God met him in tenderness and forgiveness. He saw the token which told him that all communication between heaven and earth was not severed. The way was clear and unimpeded still. Messages of reciprocated love might pass between the Father and His sinful child, as the angels in the dream ascended and descended on the visionary ladder.—*Rev. F. W. Robertson.*

Let us not forget, however, that the angels know each saint on earth more intimately than the saints themselves are known by their nearest friends. . . . But this fact suggests another analogy between our social relationships with men and angels—namely, that as early friends, who have been acquainted with ourselves and our family history during the forgotten days of infancy, are met by us, in after years, not as strangers, but with feelings of intimacy and sympathy akin to those awakened by old kindred; even so will the saint, on reaching heaven, find God's angels to be, not strangers, but old friends who have known all about him from the day of his birth until the hour of his death.—*Dr. Norman Macleod.*

## Figures and Tables

**Figure f1:**
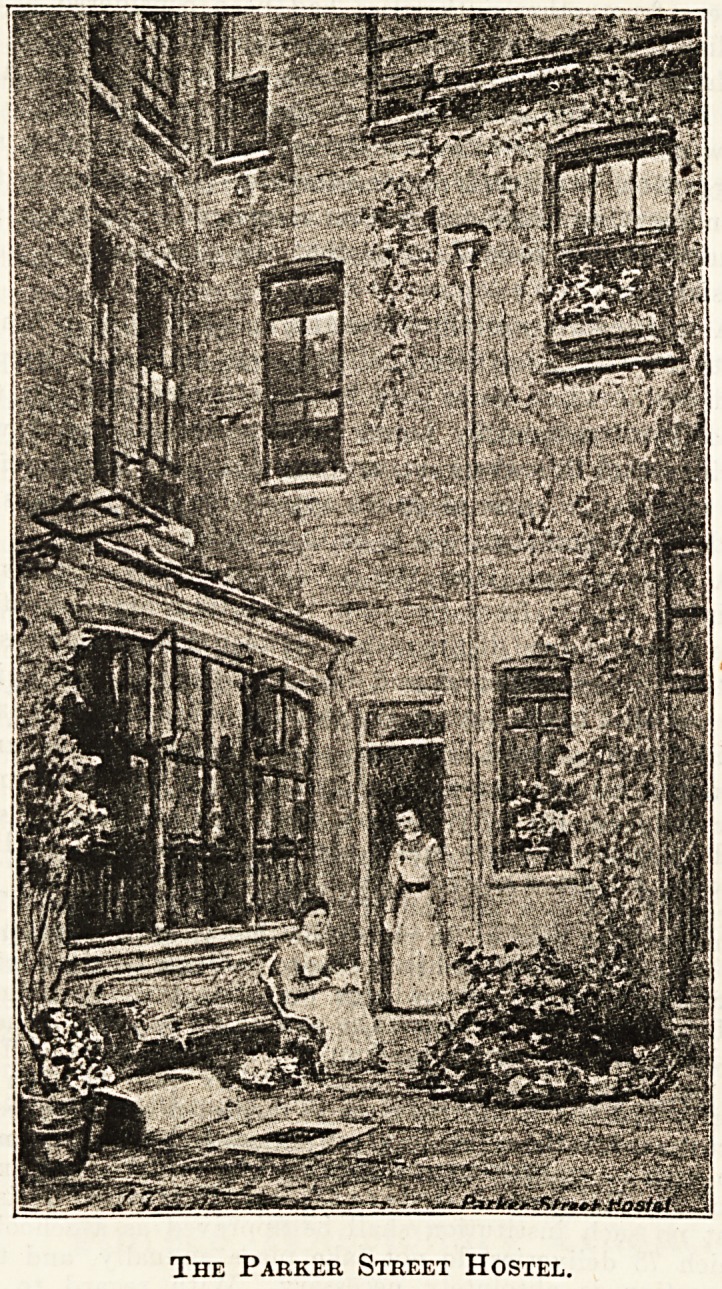


**Figure f2:**